# Anterior vs. Posterior Bite Raisers: Assessment of Quality of Life and Pain Experience

**DOI:** 10.3390/children12081040

**Published:** 2025-08-08

**Authors:** Francesca Silvestrini-Biavati, Andrea Abate, Elis Kola, Maria Elena Grecolini, Valentina Lanteri, Alessandro Ugolini

**Affiliations:** 1 Department of Surgical Sciences and Integrated Diagnostics, University of Genoa, 16126 Genoa, Italy; silvestrini.fra@gmail.com (F.S.-B.); andreabate93@gmail.com (A.A.); alessandro.ugolini@unige.it (A.U.); 2 Private Practice in Genoa, 16100 Genoa, Italy; eliskola28@gmail.com; 3 Surgical, Medical, and Dental Department, University of Modena and Reggio Emilia, 41124 Modena, Italy; dott.elena@grecoliniortodonzia.it

**Keywords:** bite-raising, build-ups, quality of life, pain, discomfort, disability

## Abstract

**Objective:** This retrospective study aims to assess the impact of bite-raising on patients’ quality of life and to compare the outcomes of anterior versus posterior build-ups. **Materials and Methods:** A total of 94 young adolescents treated with fixed orthodontic were selected and divided into two groups: the anterior bite raisers group (ABG) and the posterior bite raisers group (PBG). To enable comparison with untreated individuals, a control group (CG) of 50 subjects was also included. Pain intensity was assessed using a Visual Analog Scale (VAS), while oral health-related quality of life (OHRQoL) was measured through the OHIP-14 questionnaire (Italian validated version) before treatment and during each appointment for the first 3 months after build-ups placement. **Results:** Patients undergoing orthodontic treatment without bite raisers (CG) reported lower OHIP-14 scores compared to those with anterior (ABG) and posterior (PBG) bite raisers. In both ABG and PBG, the most commonly reported side effects included difficulty eating, oral pain, and feelings of embarrassment in social situations—similar to those reported by the control group. However, participants in the ABG also reported challenges in pronouncing certain words. Furthermore, the ABG experienced higher levels of physical pain, physical disability, and psychological discomfort compared to both the PBG and CG. Patients in the ABG reported more build-ups detachments or breakages than patients in PBG (ABG 32% vs. PBG 18%, *p* < 0.01). Build-ups were removed due to adequate overbite correction significantly before in the ABG (4.2 ± 0.9 months) than in the PBG (6.1 ± 1.4 months, *p* < 0.01). **Conclusions**: Anterior bite raisers have a significantly greater impact on patients’ quality of life compared to posterior bite raisers, leading to increased difficulties in eating and speech, higher levels of physical pain and disability, greater psychological discomfort, and more intense pain following build-ups placement.

## 1. Introduction

Temporary bite-raising, or bite-opening, is frequently required in patients undergoing orthodontic treatment. This approach facilitates the correct placement of brackets during the initial phase of treatment by keeping specific groups of teeth out of occlusion, thereby preventing bracket detachment during intercuspation. Bite-raising also removes occlusal interferences, allowing unobstructed dental movements.

Bite-raising can be achieved by applying a light-cured orthodontic resin (TRANSBOND™ PLUS 3M UNITEK^®^, 3M, St. Paul, Minnesota, USA) to the chewing surfaces of the molars or to the palatal surfaces of the front teeth.

Numerous studies in the literature discuss the impact of bite-raising on the electromyographic (EMG) activity of the superficial masseter and anterior temporalis muscles [1,2,3]; on the contrary, other clinical aspects such as chewing performance and patients’ pain and discomfort have not been investigated yet.

Pain represents a significant component of oral health-related quality of life [4,5,6,7]. Understanding how patients’ experiences of pain influence their quality of life during orthodontic treatment is very important. Orthodontic pain is a common concern for parents and patients, serving as a primary deterrent to treatment and a common cause for therapy discontinuation [8,9].

Many questionnaires have been developed to evaluate oral health-related quality of life (OHRQoL) across four key areas: oral symptoms, functional limitations, emotional well being, and social well being. These tools specifically explore the connection between pain and OHRQoL in patients undergoing treatment with fixed orthodontic appliances [8,9]. No study has focused on the investigation of the OHRQOL in patients with anterior or posterior build-ups yet.

This clinical trial aims to assess the impact of bite-raising on patients’ quality of life and to compare the outcomes of posterior versus anterior build-ups.

## 2. Materials and Methods

The present retrospective study was conducted in three orthodontic private practices located in the northwest of Italy. All participants provided written informed consent and authorized the use of their medical records for research purposes. Patient confidentiality was rigorously protected. Additionally, all clinical procedures involving human subjects reported in this study were conducted following national regulations and the principles outlined in the updated 1975 Helsinki Declaration.

The sample size for the two independent study groups (ABG and PBG) was determined based on data from a preliminary pilot sample of 10 patients, using a net increase of 1 point in the overall OHIP-14 score as the primary outcome. To achieve a power (1-β) of 0.9 with a significance level (α) of 0.05, a minimum of 47 subjects per treatment group was required.

Ninety-four subjects who were treated with fixed orthodontics and with complete VAS and OHIP records, were selected from the patient archive and divided into two groups: the anterior bite raisers group (ABG), where resin build-ups were applied to the palatal surfaces of the upper central incisors, and the posterior bite raisers group (PBG), where resin build-ups were placed on the supporting cusps of the first or second upper/lower molars. The ABG consisted of 47 subjects (22 males and 25 females; mean age 15.4 ± 1.9 years), while the PBG included 47 subjects (24 males and 23 females; mean age 15.1 ± 2.3 years).

All subjects had Angle Class I or Class II dental malocclusion, with overbite reduction and no vertical space for adequate lower brackets placement; moreover, they presented cervical vertebral maturation stage 4 or 5. Subjects presenting periodontal diseases (periodontal pocket depth > 3 mm), untreated cavitated dental lesions, maxillary overjet exceeding 5 mm (not suitable for anterior build-ups placement), presence of TMJ sounds (clicking, tripping noises, and crepitus), craniofacial syndromes, or systemic diseases were excluded from the study.

From the same archive, a control group (CG) was included to facilitate comparison of the effects of the bite raisers. The CG included 50 subjects (23 males and 27 females, age 15.5 ± 2.1 years), who followed the same orthodontic clinical protocol of the treated groups, but with no need for build-ups.

The fixed orthodontic appliances included stainless steel brackets and molar tubes placed on both the maxillary and mandibular arches, following the 0.022 inch MBT prescription. During the initial appointment, brackets and tubes were bonded to the maxillary teeth, while those to the mandibular teeth and bite raisers (in APG and PBG) were bonded during the second appointment. A sequence of NiTi and Stainless Steel (from 0.12-inch to 0.019 × 0.025-inch) wires was used. Patients attended monthly appointments, and the bite raisers were removed once the overbite correction was deemed sufficient to avoid detachment of the lower brackets. The thickness of resin build-ups was checked at each appointment and eventually increased to maintain at least 2 mm of disclusion between the upper teeth and the brackets in the lower arch.

All patients followed the same orthodontic clinical protocol, except for the build-ups placement, which was different in location between ABG and PBG and absent in CG.

During the treatment, the patients have routinely completed a Visual Analog Scale (VAS) [10,11] to assess pain levels and a questionnaire on OHRQoL using the Italian-validated OHIP-14 version [12].

The IOHIP-14 is a questionnaire composed of 14 items that assess the possible effects of dental, oral, or denture-related issues across seven specific domains (see Table 1). Participants were asked to indicate how often they experienced difficulties related to oral function, using a Likert-type scale that was converted into a Guttman scale: 0 = never, 1 = hardly ever, 2 = occasionally, 3 = fairly often, and 4 = very often. The overall unweighted IOHIP-14 score was obtained by adding the values from all 14 responses. Similarly, scores for each subscale were calculated by summing the responses corresponding to the items within that domain. Consequently, an individual’s IOHIP total score could range from 0 to 14 [12].

All participants recorded their pain levels using a 10 cm Visual Analog Scale (VAS) [10,11], with endpoints labeled ‘No pain perceived’ and ‘Worst pain imaginable’. Pain intensity was assessed at 3 and 7 days following each appointment during the first 4 months of treatment. The VAS used for this evaluation was anchored by the descriptors ‘No Discomfort’ and ‘Worst Discomfort’.

The VAS was administered through two mobile apps available on Android (https://play.google.com/store/apps/details?id=com.algos.painometerv3&hl=en (accessed on 14 May 2025)) or IOS (https://apps.apple.com/th/app/vas-visual-analog-scale/id1241222362 (accessed on 14 May 2025) platform [13]. The dental assistant sent an alert to patients to receive the screenshots with the results of their VAS via email or WhatsApp. The results of the VAS were then recorded in the database. The Italian version of the Oral Health Impact Profile-14 (IOHIP-14) questionnaire [12] (Table 1) was compiled before treatment and during each appointment for the first 4 months of therapy (for the first 3 months after build-ups placement) for a total of 5 measurements: T0 pre-treatment stage, T1 brackets placement on the upper teeth (30 days after T0), T2 build-ups placement (30 days after T1), T3 brackets placement on the lower teeth (30 days after T2), and T4 follow-up with full fixed orthodontic appliance and build-ups (30 days after T3).

Prior to initiating orthodontic treatment, all patients underwent routine screening for signs and symptoms of temporomandibular disorders (TMD). The clinical examination was performed by a calibrated senior examiner (AU), following the guidelines outlined in the RDC/TMD criteria [14,15]. In brief, the presence of temporomandibular joint (TMJ) sounds, such as clicking and/or crepitus, was evaluated through both palpation (with fingers placed over the joint area) and auscultation during mandibular opening and closing movements. The joint’s range of motion was also assessed by measuring mouth opening and observing movement patterns. This evaluation was conducted at baseline and repeated at time point T4.

### Statistical Analysis

The reliability assessment of responses to the OHIP-14 questionnaires was carried out with the intra-class correlation coefficient analysis (ICC). Internal consistency of OHIP-14 was 0.82 and the intra-class reliability coefficient was 0.91. Also, the Cronbach’s Alpha value for OHIP-14 was 0.93. The ICC for VAS measurements was 0.95. Additionally, paired *t*-tests revealed no evidence of systematic error.

A one-way analysis of variance (ANOVA) was used to analyze significant differences among groups at baseline. When a statistically significant difference was found, a post hoc test (Bonferroni corrected) was carried out.

The odds ratio (adjusted for age and gender) and 95% confidence intervals for the 14 questions of the OHIP were calculated to identify the probability that a treatment (anterior or posterior builds-up) will impact a specific area of quality of life. A probability value of *p* ≤ 0.05 was considered statistically significant in all analyses.

## 3. Results

No significant differences were observed between groups in the baseline OHIP-14 and VAS scores. In our sample, the answers “very often” and “fairly often” to questions number 3, 4, 5, 7, 8, and 10 were significantly associated with orthodontic treatment without build-ups.

Similar significant impact on OHRQoL among groups was reported for question 3, 5, and 10, while the association for question number 4 (Have you found it uncomfortable to eat any foods?), 7 (Has your diet been unsatisfactory?), and 8 (Have you had to interrupt meals?) appeared stronger for patients with build-ups, and mostly in the ABG.

**Figure 1:**
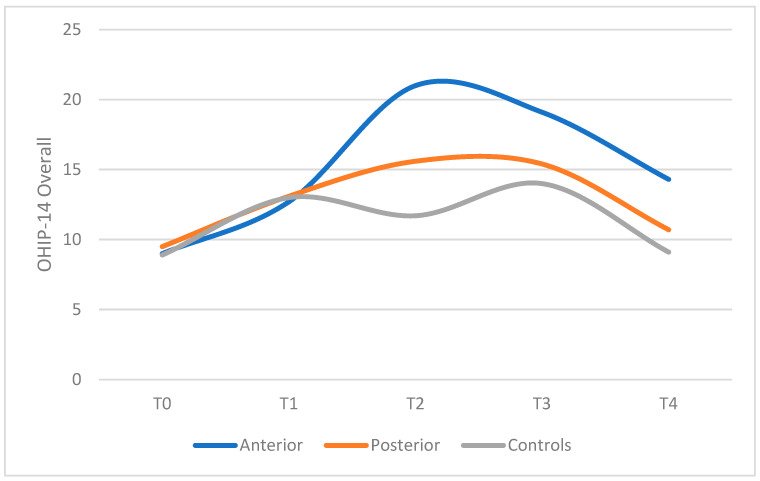
The figure represents the overall column of Table 2, i.e., the sum value of all the questions of OHIP-14.

The ABG exhibited higher levels of physical pain, physical disability, and psychological disability compared to both the PBG and CG (Table 2 and Figure 1).

The presence of anterior build-ups was also related to the increasing trouble in pronouncing some words (Adj.odds 2.66, CI = 1.68–4.22) (Table 3).

**Figure 2:**
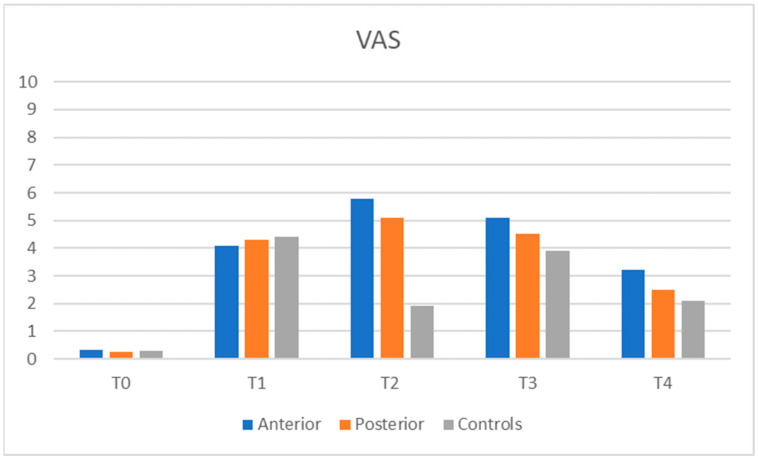
The figure represents the VAS pain scale.

Overall pain intensity was reported as low to moderate across all groups (Figure 2). However, at T2—following the placement of bite raisers—a subset of patients, particularly in the ABG, reported high pain levels. At this time point, pain intensity in both the ABG and PBG was significantly higher than in the control group and comparable to levels observed after upper (T1) and lower (T3) brackets placement. By the fifth month of treatment, no significant differences in pain intensity were observed among the groups.

Build-ups were removed due to adequate overbite correction significantly before in the ABG (4.2 ± 0.9 months) than in the PBG (6.1 ± 1.4 months, *p* < 0.01). Patients in the ABG reported more build-ups detachment or breakages (ABG: 18 subjects, 37% vs. PBG: 8 subjects 17%, *p* = 0.02). The prevalence of TMJ sounds after palpation was recorded at T4 was low and did not differ among groups (ABG: 2 subjects, 4.2%; PBG: 1 subject, 2.1%; CG: 2 subjects, 4%).

## 4. Discussion

Pain plays a critical role in the orthodontic treatment experience, significantly influencing patients’ quality of life. It is a frequent concern shared by patients, their families, and healthcare providers. The literature consistently identifies pain as a key barrier to initiating orthodontic therapy and a common factor contributing to treatment dropout: pain can influence patients’ compliance and cooperation, affecting the final outcome [16].

All orthodontic procedures, such as brackets placement, archwires activation, and application of orthopedic forces, may cause functional restrictions, discomfort, and pain. Pain is a subjective response and shows large individual variations [8]. Different questionnaires have been developed to evaluate the OHRQoL among patients wearing fixed orthodontic appliances. The original 49-item OHIP (Oral Health Impact Profile) was developed by Locker and Slade [17]. Later, to facilitate its use in a clinical setting, the questionnaire was shortened to 14 items (OHIP-14). The validity and reliability of this shortened version have been demonstrated in previous studies [18]. The OHIP-14 questionnaire was translated from English into Italian, and its validity has been confirmed; it is considered a reliable and appropriate tool for assessing the OHRQoL in the Italian population [12].

Another instrument that can be used to investigate some aspects of the OHRQoL is a Visual Analogue Scale (VAS). The VAS is a tool designed to assess subjective characteristics or attitudes—such as a patient’s perceived level of pain—that exist along a continuum and are not easily quantifiable through direct measurement [10]. It has demonstrated strong validity, high reliability, moderate responsiveness based on distribution metrics, and good responsiveness when evaluated with anchor-based methods. Compared with multi-item questionnaires, the VAS is a practical and effective option, and its use is recommended in clinical trials for evaluating overall quality of life [11,19].

In this clinical trial, both the Italian version of the OHIP-14 questionnaire and the Visual Analog Scale (VAS) were employed to assess the impact of fixed orthodontic appliances on patients’ quality of life, in particular focusing on the effect that anterior and posterior build-ups may have on OHRQoL.

Malocclusion has long been recognized as being closely linked to diminished OHRQoL. While OHRQoL may temporarily decline during orthodontic treatment, it often shows substantial improvement upon completion. Studies have also shown a relationship between OHIP scores and the clinical stage of malocclusion, particularly among groups with varying Angle classifications [20]. The esthetic improvements achieved through orthodontic treatment are closely linked to enhancements in psychological well being. Significant reductions in psychological discomfort and disability have been documented in patients undergoing comprehensive orthodontic therapy. However, the pattern of these psychological benefits varies depending on the type of malocclusion: individuals with Class I malocclusion experienced notable improvements primarily after the alignment and leveling phase, whereas those with Class III malocclusion showed consistent psychological gains throughout all treatment stages; in contrast, patients with Class II malocclusion reported the greatest benefits during the space closure phase. Then, decreases in OHIP scores can be registered at different moments of the orthodontic treatment in patients with different malocclusions. Patients included in this clinical trial had the same type and gravity of malocclusion; therefore, the malocclusion did not interfere with the OHIP scores comparison among groups and did not represent a cause of bias.

In our study, no significant differences were found among groups in baseline OHIP-14 and VAS scores (ANOVA OHIP-14 *p* = 0.23; ANOVA VAS *p* = 0.41). All subjects had homogeneous characteristics: Angle Class I or Class II dental malocclusion with overbite reduction and no vertical space for an adequate lower brackets placement. All patients followed the same orthodontic clinical protocol, except for the build-ups placement, which was different in location between ABG and PBG, and absent in CG.

OHIP-14 questionnaire (Table 1) was completed before treatment and then, during each appointment for the first 4 months of therapy. Data were collected in five moments: T0 pre-treatment stage, T1 brackets placement on the upper teeth, T2 build-ups placement, T3 brackets placement on the lower teeth, T4 follow-up with a full fixed orthodontic appliance and build-ups. Overall OHIP-14 scores were calculated by summing the responses to each item (Table 2), with higher scores indicating poorer OHRQoL. The results demonstrated that both at the start of orthodontic treatment and at each appointment involving the placement of a new orthodontic device, there was a temporary but significant increase in OHIP-14 scores. It is generally recognized that insertion of a fixed appliance worsens patients’ OHRQoL; the most frequent complaints are impaired speech, impaired swallowing, feeling of oral constraint, and lack of confidence in public.

Patients receiving orthodontic treatment without bite raisers (CG) exhibited lower OHIP-14 scores compared to those in the ABG and PBG. The multibrackets appliance alone did not affect in a significant way patients’ ability to pronounce any words, nor the sense of taste; no negative effects were reported on patients’ mood, behavior, and working ability. The most commonly reported side effects of orthodontic treatment were difficulty eating, oral pain, and feelings of embarrassment in social settings.

Compared to T0, overall OHIP significantly increased in T1, T2, and T3 after bonding the brackets on the upper and lower teeth, while it decreased in T4 during follow-up, probably because patients got used to the orthodontic appliance. Therefore, although patients gradually adapted to the physical effects of treatment, feelings of self-consciousness and embarrassment remained significantly prevalent throughout therapy, highlighting the substantial psychosocial impact of orthodontic treatment on patients’ lives.

Patients receiving orthodontic treatment with either anterior or posterior bite raisers (ABG, PBG) exhibited higher OHIP-14 scores from the time of build-ups placement compared to the control group (CG). In both ABG and PBG, difficulty eating, oral pain, and embarrassment in public were the most frequently reported side effects, similar to those in the CG. However, patients in the ABG also reported difficulties in pronouncing certain words.

Table 2 represents the Odds Ratio (OR), a statistical measure that quantifies the strength of association between two variables, often used in epidemiology and clinical research. In essence, it expresses the ratio between the probabilities of an event in two groups, one exposed and one not exposed to a certain condition. In this study, it was observed that patients in the ABG had a 2.66-fold increased risk of experiencing speech problems compared to those in the PBG or CG, with an Adj. OR of 2.66 * (1.68–4.22); all patients had a significantly increased risk of developing mouth pain and difficulty eating, while patients with ABG showed a greater risk of developing psychological discomfort and disability with an Adj. OR of 1.48 * (0.89–169) (question 6) and of 1.99 * (1.10–3.59) * (question 10).

The comparison between anterior and posterior build-ups showed that anterior build-ups increased the OHIP-14 scores more than posterior build-ups: patients in ABG reported many more difficulties in eating and speech than patients in PBG; the ABG exhibited higher levels of physical pain, physical disability, and psychological disability. The intensity of pain at T2 was higher for ABG than PBG and significantly greater with respect to CG; after bite raisers placement, a small number of subjects, mainly in the ABG, reported experiencing high levels of pain. Pain values after positioning the anterior or posterior build-ups were comparable to those registered after upper (T1) and lower (T3) brackets placements. Build-ups placement had a similar impact on patients OHRQoL as upper or lower brackets placement.

Since our clinical protocol contemplated the positioning of upper brackets, lower brackets, and eventually bite raisers within three months, OHIP-14 scores were high in the first phase of the orthodontic treatment, with a progressive worsening of patients’ OHRQoL during the positioning of all the orthodontic appliances. Then, starting from T4, OHIP-14 scores slowly decreased, probably because patients adapted to the different devices.

Patients in the ABG reported more build-ups detachments or breakages than patients in PBG (ABG 32% vs. PBG 18%, *p* < 0.01). Build-ups were removed due to adequate overbite correction significantly before in ABG (4.2 ± 0.9 months) than in the PBG (6.1 ± 1.4 months, *p* < 0.01); anterior build-ups appeared more effective in the overbite correction than posterior build-ups, but they required more adjustments by the clinician, and they were more annoying for patients.

The prevalence of TMJ sound or TMJ pain after palpation recorded at T1 and T4 was low and did not differ among groups (ABG 3.1%; PBG 2.8%; 2.3% CG). This finding is in accordance with the literature, which shows no scientific evidence supporting a causal relationship between orthodontic treatment and temporomandibular disorders (TMD) [14].

In the literature, it has been postulated that patients with high catastrophizing scores in the early stages experience more intense pain during orthodontic treatment with fixed appliances [21]. In our article, we did not investigate this component, and this may represent a limitation of the study, as it could be a confounding factor that may have influenced the responses to some of the questionnaire items.

## 5. Conclusions

- Anterior bite raisers have a considerably greater impact on patients’ quality of life compared to posterior bite raisers, resulting in more pronounced difficulties with eating and speech, increased physical pain, physical disability, psychological disability, and higher pain intensity following build-ups placement.
- Anterior build-ups appear more effective in the overbite correction than posterior build-ups, but they require more adjustments by the clinician, and they are more annoying for patients.
- The use of bite raisers does not affect the prevalence of TMJ sound or TMJ pain after palpation, independent of their location (anteriorly or posteriorly).
- In patients who are particularly sensitive to pain, it is better to postpone the placement of the build-ups by a month after brackets placement.

## Figures and Tables

**Table 1:** The table represents the Oral Health Impact Profile-14 (IOHIP-14) questionnaire.

OHIP-14 Scores		
**Functional** **limitation**	Have you had trouble pronouncing any words?	1
	Have you felt that your sense of taste has worsened?	2
**Physical** **pain**	Have you had painful aching in your mouth?	3
	Have you found it uncomfortable to eat any foods?	4
**Psychological** **discomfort**	Have you been selfconscious?	5
	Have you felt tense?	6
**Physical** **disability**	Has your diet been unsatisfactory?	7
	Have you had to interrupt meals?	8
**Psychological** **disability**	Have you found it difficult to relax?	9
	Have you been a bit embarrassed?	10
**Social** **disability**	Have you been a bit irritable with other people?	11
	Have you had difficulty doing your usual jobs?	12
**Handicap**	Have you felt that life in general was less satisfying?	13
	Have you been totally unable to function?	14

**Table 2:** The table highlights the average response to the questionnaire represented in Table 1.

		Functional Limitation	Physical Pain	Psychological Discomfort	Physical Disability	Psychological Disability	Social Disability	Handicap	Overall
T0	Anterior	1.2	1.7	1.9	1.3	1.4	0.7	0.8	9
	Posterior	1.5	1.8	2.2	0.9	1.6	0.9	0.6	9.5
	Control	1.3	1.5	1.9	1.4	1.2	0.9	0.7	8.9
T1	Anterior	2	2.4	2.1	1.6	2.2	1.2	1.2	12.7
	Posterior	1.9	2	2.4	2.1	1.8	1.5	1.4	13.1
	Control	1.9	1.9	2.2	2	2.1	1.4	1.5	13
T2	Anterior	3.1	4.8	2.9	3.7	2.5	2.3	1.7	21
	Posterior	2	3.6	2.5	2.6	1.8	1.7	1.4	15.6
	Control	1.6	2	2.2	1.5	1.6	1.7	1.1	11.7
T3	Anterior	3.2	4.3	2.7	3.2	2.2	2.2	1.3	19.1
	Posterior	2.2	2.7	2.4	2.5	2.3	2.1	1.2	15.4
	Control	1.7	2.5	2.3	1.9	2.1	2.3	1.2	14
T4	Anterior	2.5	3.1	1.9	2.1	1.8	1.7	1.2	14.3
	Posterior	1.4	1.9	1.6	1.8	1.5	1.4	1.1	10.7
	Control	1.1	1.5	1.8	1.4	1.2	1.3	0.8	9.1

**Table 3:** In the table, the results of the previous table are compared, and the statistically significant ones are indicated with the symbol “*”.

OHIP-14 Scores			Anterior	Posterior	Ortho
			Adj. OR (95%CI)	Adj. OR (95%CI)	Adj. OR (95%CI)
**Functional limitation**	Have you had trouble pronouncing any words?	1	2.66 * (1.68–4.22) *	1.10 (0.74–1.63)	1.40 (0.97–2.02)
	Have you felt that your sense of taste has worsened?	2	1.28 (0.86–1.93)	1.09 (0.65–1.84)	0.74 (0.43–1.29)
**Physical pain**	Have you had painful aching in your mouth?	3	2.28 * (1.37–3.78)	2.85 * (2.13–3.82) *	2.50 * (2.04–3.07) *
	Have you found it uncomfortable to eat any foods?	4	4.38 * (3.09–6.22) *	2.78 * (2.05–3.80)	1.67 * (1.25–2.23)
**Psychological discomfort**	Have you been self-conscious?	5	2.01 * (1.22–3.30) *	1.56 * (1.38–1.76)*	1.78 * (1.31–2.43) *
	Have you felt tense?	6	1.48 * (0.89–1.69)	1.30 (0.95–1.79)	1.55 (0.77–3.13)
**Physical disability**	Has your diet been unsatisfactory?	7	4.47 * (1.78–11.23) *	2.78 (2.05–3.80) *	1.67 (1.25–2.23) *
	Have you had to interrupt meals?	8	3.71 * (1.82–7.57) *	1.82 (1.30–2.56) *	1.58 (1.25–1.98) *
**Psychological disability**	Have you found it difficult to relax?	9	0.92 (0.42–1.95)	0.71 (0.33–1.47)	1.23 (0.89–1.72)
	Have you been a bit embarrassed?	10	1.99 * (1.10–3.59) *	2.23 (0.81–6.17)	2.61 * (1.97–3.16) *
**Social Disability**	Have you been a bit irritable with other people?	11	0.97 (0.69–1.35)	1.18 (0.83–1.66)	1.30 (0.61–2.89)
	Have you had difficulty doing your usual jobs?	12	0.74 (0.50–1.09)	0.75 (0.51–1.12)	0.91 (0.71–1.17)
**Handicap**	Have you felt that life in general was less satisfying?	13	0.62 (0.39–0.99)	0.83 (0.53–0.99)	0.95 (0.69–1.32)
	Have you been totally unable to function?	14	0.45 (0.19–1.01)	0.54 (0.19–1.00)	0.59 (0.29–1.30)

## Data Availability

The raw data supporting the conclusions of this article will be made available by the authors on request because patients involved in the study have been treated in private practice.
